# Automated systems to identify relevant documents in product risk management

**DOI:** 10.1186/1472-6947-12-13

**Published:** 2012-03-02

**Authors:** Xue Ting Wee, Yvonne Koh, Chun Wei Yap

**Affiliations:** 1 Department of Pharmacy, National University of Singapore, 18 Science Drive 4, Singapore 117543, Republic of Singapore; 2 Vigilance Branch, Vigilance, Compliance & Enforcement Division, Health Products Regulation Group, Health Sciences Authority, 11 Biopolis Way, Helios, Singapore 138667, Republic of Singapore

## Abstract

**Background:**

Product risk management involves critical assessment of the risks and benefits of health products circulating in the market. One of the important sources of safety information is the primary literature, especially for newer products which regulatory authorities have relatively little experience with. Although the primary literature provides vast and diverse information, only a small proportion of which is useful for product risk assessment work. Hence, the aim of this study is to explore the possibility of using text mining to automate the identification of useful articles, which will reduce the time taken for literature search and hence improving work efficiency. In this study, term-frequency inverse document-frequency values were computed for predictors extracted from the titles and abstracts of articles related to three tumour necrosis factors-alpha blockers. A general automated system was developed using only general predictors and was tested for its generalizability using articles related to four other drug classes. Several specific automated systems were developed using both general and specific predictors and training sets of different sizes in order to determine the minimum number of articles required for developing such systems.

**Results:**

The general automated system had an area under the curve value of 0.731 and was able to rank 34.6% and 46.2% of the total number of 'useful' articles among the first 10% and 20% of the articles presented to the evaluators when tested on the generalizability set. However, its use may be limited by the subjective definition of useful articles. For the specific automated system, it was found that only 20 articles were required to develop a specific automated system with a prediction performance (AUC 0.748) that was better than that of general automated system.

**Conclusions:**

Specific automated systems can be developed rapidly and avoid problems caused by subjective definition of useful articles. Thus the efficiency of product risk management can be improved with the use of specific automated systems.

## Background

Product risk management involves critical assessment of health product safety issues by evaluating their risk-benefit ratio, on occasion followed by taking appropriate regulatory actions to mitigate these safety concerns [1]. Environmental scanning of safety information of health products is usually conducted through several sources to ensure thorough search and extensive coverage. Firstly, local trends of adverse events incidence can be obtained from spontaneous adverse events reporting. These adverse events are observed during clinical practice and reported to the regulatory agency by healthcare professionals or by pharmaceutical manufacturers. Information on product safety may also be obtained from safety alerts disseminated by different regulatory authorities such as the Therapeutic Goods Administration in Australia, the Food and Drug Administration in the United States of America and the European Medicines Agency in Europe. Regulatory authorities all over the world work closely together and alert each other of any safety concerns raised. Another source of safety information will be the primary literature where results of clinical trials, case reports and other safety-related studies may be reported.

Primary literature remains as a valuable source of drug safety information, especially for newer drugs where there is little regulatory experience with them. With intensive research in medical sciences, primary literature is generated at a very rapid rate. In PubMed alone, more than 600,000 articles are added each year from over 5000 journals [2]. Although, there is voluminous amount of information available, only a small portion is useful for risk assessment. For instance, a search in PubMed for tumour necrosis factor-alpha (TNF-α) blockers using the search terms 'adalimumab', 'infliximab' and 'etanercept' in Medical Subject Headings (MeSH) [3] retrieved close to 4000 articles. However, only about 700 (17.5%) contain valuable information for risk assessment work. Thus, it is time-consuming and inefficient to manually sieve through this large number of articles and identify those that are valuable to product risk assessment. Hence, the ability to expedite this process of useful literature identification can contribute to risk assessment efficiency.

### Text mining as a potential solution

Text mining is defined as the process of retrieving or extracting small nuggets of relevant information from large collections of textual data [4]. It is a powerful tool to identify word usage patterns and has already been effectively deployed in many areas such as email classification [5,6], legal/business applications [7,8] and biomedical text analysis[9,10]. In one study, Agarwal and Yu had shown that the use of text mining was able to achieve 91.95% of accuracy in automatically classifying sentences from biomedical full text into introduction, methods, discussion and conclusion categories [11]. Wang et al showed that text mining was able to achieve 95% sensitivity and specificity in 51.5% of abstracts that were automatically classified for the purpose of Immune Epitope Database [12]. Recently, text mining was used to improve systematic reviews of adverse drug reactions by identifying such articles from medical literature with a recall of 70% and precision of 21% [13]. Hence, text mining had been shown to be a powerful and useful tool in the automated classification of textual data. It will be interesting to explore text mining as a potential solution for developing an automated system to identify relevant documents for product risk management work.

In this work, two automated systems were developed and explored for their usefulness in identifying 'useful' articles from their titles and abstracts (henceforth referred to as just abstracts) in the PubMed database. The first system used only general terms that were found in the abstracts as predictors and thus is able to identify 'useful' articles regardless of the drug class. However such general automated system may not have sufficiently high accuracy for routine risk assessment work. Thus, a second system which was specific for a particular drug class was developed and tested. During routine work, evaluators will manually classify a small number of articles related to the drug class of interest. The second system will then learn from the abstracts of these articles and develop a model that is specific for that drug class.

In order to develop the two automated systems, large amounts of journal articles have to be manually classified. Ideally, a large number of articles from different drug classes should be used, especially for the development of the general automated system. However, it is tedious and impractical to perform manually classification of such a large number of articles. Thus, in this study, only articles on TNF-α blockers were manually classified and used to develop the two automated systems. TNF-α blockers were chosen because they have a relatively small corpus of literature, which make it suitable for manual classification.

TNF-α blockers are biologics that are indicated for several autoimmune diseases such as psoriasis, rheumatoid arthritis and Crohn's disease, and are playing an emerging role under circumstances when these disease conditions are refractory to conventional therapies [14]. However, intensive post-marketing surveillance [15,16] and case reports [17-19] reveal rare but severe adverse effects such as opportunistic infections, reactivation of latent infections, new-onset psoriasis and lymphomas. It is unsure whether these adverse effects are due to predisposition by the underlying diseases or the adverse effects of TNF-α blockers per se, therefore post-marketing surveillance is of paramount importance in monitoring the safety profile this group of drugs.

## Results

### Comparison of performance of various algorithms

Table 1 shows the performance of various algorithms on the validation set. The results suggested that the model developed using SVM had the best prediction performance compared to the models developed using other learning algorithms. Thus, SVM was chosen for the development of the general and specific automated systems.

**Table 1:** Comparison of performance of different algorithms using general predictors on the validation set

Model	AUC
Logistic regression	0.829
K-nearest neighbor (k = 3)	0.642
Naive Bayes	0.673
SVM (gamma = 1.0, C = 0.0)	0.870

### Comparison of performance of different types of frequencies

Table 2 shows the comparison of performance of SVM models trained using different types of frequencies on the validation set. SVM models developed using TF-IDF had better prediction performance compared to those developed using either word occurrence or binary frequency. Thus, the general and specific automated systems were developed using TF-IDF values.

**Table 2:** Comparison of performance of SVM models using different types of frequencies on the validation set

Type of frequency	All predictors	General predictors
Word occurrence	0.892	0.870
Binary frequency	0.849	0.828
TF-IDF	0.909	0.898

### General automated system

**Figure 1:**
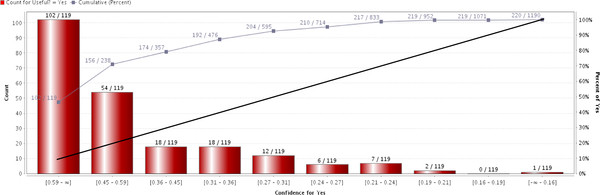
**Lift chart of SVM model trained using TF-IDF of general predictors on validation set**.

Figure 1 shows the lift chart of the general automated system developed using SVM and TF-IDF of general predictors on the validation set.

The black line shows the cumulative percentage of 'useful' articles with the assumption that 'useful' papers would be distributed at random in each 10% sample of the set. This represents the case where the general automated system was not used. In such a scenario, identification of 'useful' papers will follow a linear progression correlated to the total number of examined papers.

The line graph shows the cumulative percentage of 'useful' articles when the general automated system was used to present papers to the evaluators in decreasing order of confidence. The confidence values were computed by the SVM model for each article. The percentages for both the black line and line graph can be read using the vertical axis on the right. For instance, 46.4% (102 out of 220) and 70.9% (156 out of 220) of the total number of 'useful' articles were among the first 10% and 20% of the articles presented to the evaluators by the general automated system respectively. The difference between the line graph and black line shows that the general automated system was able to improve the identification of 'useful' articles by using the SVM confidence values to rank the articles, compared to the case when the automated system was not used.

Each red bar represents the number of 'useful' articles contained in every 10% of the total articles presented to the evaluators by the system. For example, the first bar showing 102/119 shows that there were 102 'useful' articles among the first 119 articles that were presented to the evaluators by the general automated system. In the second bar, 54 'useful' articles were present among the next 119 articles.

**Figure 2:**
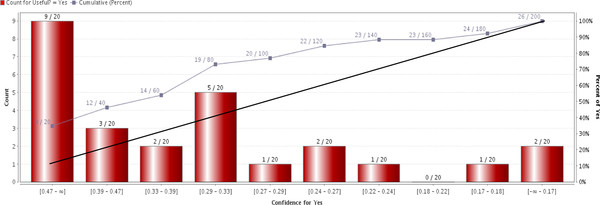
**Lift chart of SVM model trained using TF-IDF of general predictors on generalizability set**.

The general automated system was also validated using the generalizability set in order to assess its generalizability performance. The AUC was 0.731 and Figure 2 shows the lift chart for the system on generalizability set. The system was able to rank 34.6% and 46.2% of the total number of 'useful' articles among the first 10% and 20% of the articles presented to the evaluators respectively.

### Specific automated system

Table 3 shows the prediction performance of the specific automated system on the validation set when it was developed using training sets of various sizes. The results showed that the prediction performance of the specific automated system generally improves when more articles were used to develop the system. The prediction performance of the specific automated system was generally better than the generalizability performance of the general automated system when more than 20 articles were used to develop the specific automated system.

**Table 3:** Comparison of performance of models trained on various training set sizes using all predictors on the validation set

Training set size	Ratio of useful: non-useful articles	AUC
2	1: 1	0.684
20	1: 9	0.748
36	1: 17	0.699
74	1: 9.57	0.749
112	1: 4.89	0.794

## Discussion

### Comparison of performance of various algorithms

Different supervised learning algorithms were used to develop various prediction models. The results showed that the model developed using SVM had the best prediction performance compared to those developed using other algorithms. This is in concordance with other studies which frequently showed that models developed using SVM outperforms those developed using other learning algorithms [20].

### Comparison of performance using different types of frequencies

The prediction performance of models developed using either word occurrence, binary frequency or TF-IDF of predictors were compared. The model developed using TF-IDF showed the best performance, followed by the model using word occurrence and lastly the model using binary frequency. In the computation of TF-IDF, terms that occurred less frequently in the training set were given higher values and those that occurred very frequently were given lower values. A possible reason for the better prediction performance of the model developed using TF-IDF is that those words that occur less frequently play a key role in the classification of 'useful' and 'non-useful' articles. In addition, binary frequency can only provide information on the presence or absence of a term and not its occurrence frequency. Thus, the poorer performance of the model developed using binary frequency suggested that the classification of the articles may be dependent on the frequency of term occurrence.

### General automated system

A general automated system was developed using SVM and TF-IDF of general predictors. Only general predictors were used to develop the system because predictors that are specific for TNF-α blockers are usually not present in articles on other drug classes. Thus, the use of these specific predictors in an automated system will add noise to the system, which will reduce its generalizability.

The results showed that the general automated system was able to rank the articles such that approximately 70% of the 'useful' articles on TNF-α blockers were found in the first 20% of TNF-α blockers articles. The generalizability performance for classifying articles on four other drug classes were lower, which suggests that there could be other general predictors which were not present in the abstracts of articles on TNF-α blockers. Thus there is a need to periodically update the general predictors used in the general automated system with newly classified abstracts of articles from different drug classes in order to maintain or improve its generalizability performance.

The advantage of the general automated system is that the evaluators need not manually classify any articles before using the system. A major limitation of the general automated system is that the system is trained using abstracts that were manually classified into 'useful' and 'non-useful'. Although a systematic approach (Additional file 1) was used in this study to classify the articles, the classification scheme may not be applicable for different drug classes or different risk assessment tasks. In fact, it is important to note that product risk management involves using a variety of information sources and primary literature is just one of the sources. Thus different risk assessment tasks may have slightly different definition of 'useful' and 'non-useful' articles. This subjectiveness in classification of 'useful' articles may limit the applicability of the general automated system.

### Specific automated system

The general trend of the prediction performance of specific automated systems developed using training sets of various sizes is that an increase in training set size correlated with improved performance of the automated system. The AUC of the system trained using 36 articles appeared to be an anomaly from this general trend. This could be due to its ratio of 'useful' to 'non-useful' articles, which was much more unbalanced compared to those in the rest of the training sets. Studies have shown that models developed from highly unbalanced training sets tend to have poorer prediction performance than those developed from balanced training sets [21,22].

The advantage of the specific automated system is that a new automated system will be developed for each new drug class or new risk assessment task. This resolves problems caused by the subjective definition of 'useful' and 'non-useful' articles and thus prevent the potential poor generalizability associated with the general automated system. This study shows that manual classification of just 20 articles is sufficient to develop a specific automated system. Based on our experience, an experienced evaluator can easily manually classify 20 articles within a short time of approximately 30 minutes. Thus the use of such specific automated system is feasible for routine risk assessment work.

One disadvantage is that in the selection of articles for manual classification, 'useful' articles may not be selected by the Kennard and Stone sampling method or the selection may result in a highly unbalanced 'useful' to 'non-useful' articles ratio in the training set which may potentially decrease the performance of the automated system. The lower performance of the automated system trained using 36 articles could constitute an example of such problem.

A potential solution is to use the general automated system to perform the initial selection of articles that are most likely to be 'useful' and articles that are most likely to be 'non-useful' for manual classification. Although the general automated system may not have high generalizability performance, using it will still increase the likelihood of achieving a more balanced 'useful' to 'non-useful' articles ratio in the training set, compared to the alternative option of not using it.

### Potential application of automated systems in routine risk assessment work

**Figure 3:**
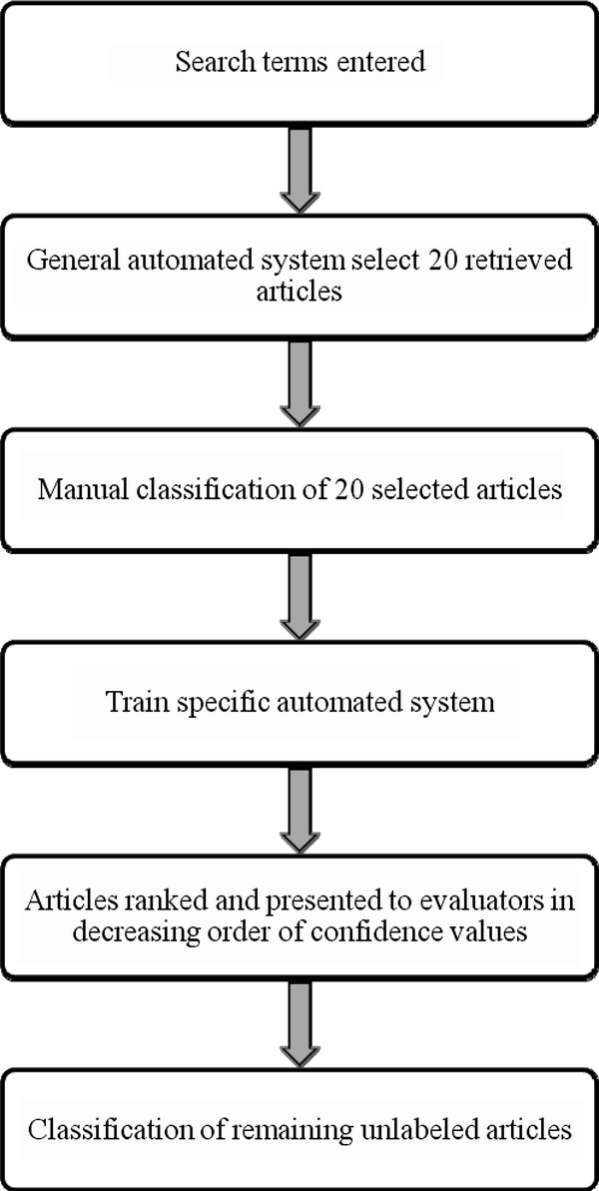
**Use of automated system in routine risk assessment work**.

During routine risk assessment work, evaluators will enter search terms into the system. The system will proceed to retrieve abstracts of articles from PubMed. The general automated system will then select a list of approximately 20 abstracts to present to the evaluators for manual classification. The specific automated system will then be trained using these manually classified articles. The specific automated system will categorize the retrieved articles using their abstracts and rank them according to the confidence values for the classification of the articles as 'useful'. The list of abstracts will be presented to the evaluator in decreasing order of confidence values. This work flow is summarized in Figure 3.

### Current limitations and potential for improving the automated systems

In this study, parameter selection of the SVM algorithm was guided by determining the AUC of the automated systems on the testing set. An implicit assumption for AUC values is that both false positives and false negatives are equally problematic. This is clearly not the case for risk assessment task as false positives will only result in additional workload for the evaluators and will be identified during manual review of the results. In comparison, false negatives may result in important articles not being examined. Thus, future studies could explore using a customized performance measure which weighs false positives and false negatives differently to guide parameter selection.

The automated systems were developed using 'useful' and 'non-useful' articles. This assumes that all 'useful' articles are equally valuable. This is an over-simplification as some articles are more 'useful' than others. However, it is difficult to determine the degree of 'usefulness' for an article and thus it is not possible to develop regression models for use in the current automated systems. Future work could attempt to address this limitation by developing methods to ease the determination of the degree of 'usefulness' for an article.

In this study, it was assumed that important information required for classification of the articles will be summarized in the abstracts. However, authors may describe safety issues in the article but did not include them in the abstracts. In addition, several articles may discuss about similar safety issues but not all will include warnings and precautions on the use of these drugs in the abstracts. Hence, not all potentially 'useful' articles could be identified by the two automated systems which use only the abstracts. A potential solution is to develop and apply the automated systems on full articles [23]. However, the presence of several obstacles made it challenging to make use of full articles in such automated system [24]. Firstly, bulk download of full articles is difficult to automate. Moreover, copyright and fair use issues make retrieval of full text of all entries indexed on PubMed unfeasible. In addition, retrieved documents are often in PDF or HTML format and will be required to be converted into plain text prior to being used for text mining. Unfortunately, this conversion is not always accurate. Furthermore, symbols (e.g. 'ε' and 'α') are frequently used in full articles of biomedical literature and they require replacement with their spelled names. Such replacements have usually been done in their abstracts, which makes preparation of abstracts for feeding into automated systems less tedious.

A potential method to improve the performance is to explore semantic features. Semantics refers to the study of 'meanings' linked to their words in linguistic studies [25]. Semantic features could be applied by using MeSH and the Unified Medical Language System (UMLS) concepts and semantic types [12,20]. UMLS is the most extensive known database of synonyms and concepts relations of biomedical and health-related terms, maintained by National Library of Medicine. It can be used to map related concepts to words in text mining applications [26] and has been used to extract disease drug knowledge from biomedical and clinical documents [27,28]. This addition of meanings and concepts of the terms linked to their respective concepts may potentially boost the performance of the model.

Another method to improve performance is to use MeSH terms in addition to the abstracts for creating the predictors. The reason for using MeSH terms is because it contained information on the topics of the articles, which may be useful for the classification of the articles by the automated systems. For example, articles related to pharmacoeconomics are often focused on the decrease in cost burden to patients and to society but not on the adverse drug reactions. On the other hand, articles related to pharmacokinetics of a drug may occasionally be useful as they may report on dose-dependent adverse drug reactions. The inclusion of MeSH terms had been found to improve the prediction performance of the epitope model [12] and thus could possibly be useful for improving the performance of the automated systems.

## Conclusions

In this study, a general automated system for identifying relevant journal articles for product risk assessment work was developed using a large set of abstracts of articles on TNF-α blockers, which were manually classified as 'useful' or 'non-useful'. In addition, the feasibility of developing a specific automated system for each drug class was explored. The results showed that the general automated system was generally able to rank 'useful' articles ahead of 'non-useful' articles, though that ability was poorer on drug classes other than TNF-α blockers. In addition, the subjectiveness in determining 'useful' and 'non-useful' articles may preclude adoption of the general automated system for routine product risk management work. In comparison, a specific automated system with better prediction performance than the general automated system could be developed using approximately 20 abstracts. Since a new specific automated system has to be developed for each new risk assessment task, it will avoid the problem that is caused by the subjective definition of 'useful' and 'non-useful' articles. Hence, the results from this study shows that text mining is suitable to improve the efficiency of literature search performed during comprehensive risk assessment. It is important to note that the use of such automated systems will not make risk management more effective but instead is to help access all data in the medical literature that is relevant to risk management reviews more effectively.

## Methods

### Data collection

Using the search terms 'adalimumab', 'infliximab' and 'etanercept' in MeSH [3], a total of 3966 abstracts of articles relevant to TNF-α blockers that were indexed on PubMed were retrieved (as of 11 September 2009). The retrieved articles undergo manual classification into 'useful' and 'non-useful' categories. In this study, 'useful' articles are defined as those containing information that may potentially warrant a regulatory action like an update on the contraindications, warnings and precautions on the use of the drug. These articles may report on newly encountered adverse events, incidences of rare adverse events, mechanisms of action of certain adverse events or any other relevant data. For example, a review that shows TNF-α blockers may possibly be causative agents in congenital abnormalities is considered as a 'useful' article [29]. 'Non-useful' articles refer to any other articles that do not contain any of the above mentioned relevant' information. Additional file 1 shows the classification algorithm used in this study to classify uncomplicated cases. For complicated and ambiguous cases, the full articles were retrieved for further review to ensure accurate classification. A total of 693 articles were classified as 'useful' (17.5%) and 3273 articles were classified as 'non-useful' (82.5%).

**Figure 4:**
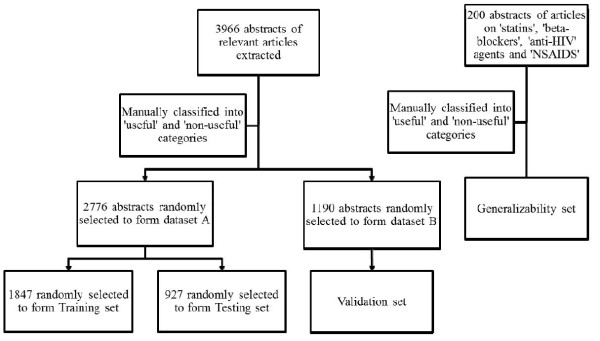
**Splitting of dataset**.

The classified data were then randomly split into two datasets, A and B, each containing 70% and 30% of the examples respectively. Dataset A was further randomly split into a training set and a testing set in a ratio of 2:1 and Dataset B was used to form the validation set. The validation set was used to determine the performance of the models and was not used during model development. Stratified random split was used in all the splitting of datasets to maintain the same proportion of 'useful' to 'non-useful' articles in all the datasets. An overview of the division of the dataset is summarized in Figure 4.

### Text mining

**Figure 5:**
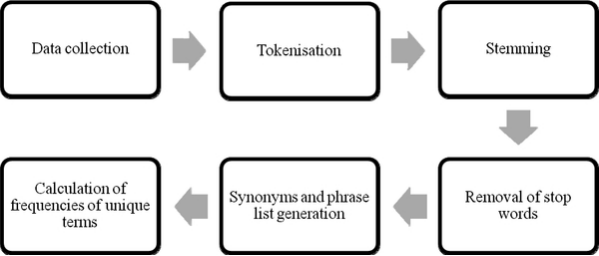
**Summary of text mining work flow**.

Text mining was independently done on the abstracts in training, testing, validation and generalizability sets using the Text Mining module in Statistica Text Miner v9.0 [30]. The module first breaks down sentences in the abstracts into individual tokens. Examples of tokens include words, acronyms, abbreviations, numbers and punctuation symbols. Next, as most content-bearing terms undergo inflection (e.g. as prepositions, adjectives or conjunctions), these terms were linked with their root terms in a process known as stemming. For instance, 'activate', 'activating', 'activated' and 'activates' will be stemmed to the root term 'active'. Stop words were then removed. Stop words referred to words that occur frequently but conveyed very little information. Most of them included prepositions such as 'while', 'to' and 'on'. The default list of stop words provided by the Text Mining module were used. Subsequently, acronyms and synonyms were identified and combined with their root forms. The list of synonyms were obtained from Omniviz [31] and includes variations of British and American English. Phrases were also identified using the list of phrases obtained from Omniviz. For example, 'tumour necrosis factor' was considered as a single term instead three separate terms. Finally, the word frequency of each term in each abstract was calculated. Figure 5 show the text mining process.

The extracted terms are also known as predictors because they are used by the models to predict the usefulness of unlabeled articles based on specific mathematical calculations. These predictors were manually classified into 'specific' and 'general' categories. Predictors that were specific to TNF-α blockers or diseases treated by TNF-α blockers were classified as specific, and the rest were classified as general predictors. The list of 730 general predictors can be found in Additional file 1. The frequency of a predictor in an abstract can be calculated as word occurrence, binary frequency or term frequency-inverse document frequency (TF-IDF). Word occurrence is the number of times a predictor occurs in an abstract. For binary frequency, the presence or absence of a predictor in an abstract was determined and recorded as '1' or '0' respectively. In the TF-IDF method, a TF-IDF*_i, j _*value corresponding to the importance of a predictor *i *in a document *j *was computed using the following:

(1) $$TF_{i,j}=\frac{n_{i,j}}{\underset{k}{\sum }n_{k,j}}$$

(2) $$IDF_{i}=\text{log}\left(\frac{N}{n_{i}}\right)$$

(3) $$TF\;\text{ - }\;IDF_{i,j}=TF_{i,j}\times IDF_{i}$$

where *n_i, j _*is the number of occurrences of predictor *i *in document *j, N *is the total number of documents and *n_i _*is the number of documents in which predictor *i *occurs. In TF-IDF, predictors that were common in an abstract *j *but were less common in all the other abstracts were considered as more important to abstract *j *[32]. These three types of frequencies were used in this study to determine the optimum type for classifying articles for risk assessment work.

### Supervised machine learning

Supervised learning methods were used in this study to develop the models as it had been shown to have better performance than unsupervised learning methods in other studies [20]. In supervised learning, the prediction model was trained to recognize patterns in the abstracts of both 'useful' and 'non-useful' articles. Supervised learning algorithms such as logistic regression [33], k-nearest neighbors [34], Naive Bayes [32] and support vector machine (SVM) [35,36] implemented in the software RapidMiner 5.0 [37] were used to develop the models. These algorithms have been extensively described elsewhere and thus only a brief description is provided for SVM, which was found to have the best performance in this study.

**Figure 6:**
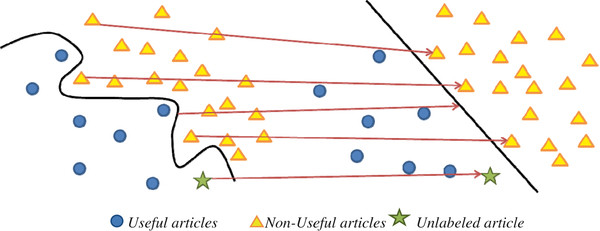
**Projection of abstracts to a high dimensional space in a SVM model**.

In SVM, 'useful' and 'non-useful' articles in the training set were mapped onto a multi-dimensional space and the coordinates of each article were determined by the TF-IDF values of the predictors in its abstract [38]. A subset of 'useful' and 'non-useful' articles that were located near each other in this multi-dimensional space was identified by SVM and these articles are known as positive and negative support vectors respectively. A decision boundary can be drawn between the positive and negative support vectors to separate the two types of articles. In most cases, the positive and negative support vectors could not be linearly separated (see left of Figure 6). Thus, a kernel function, such as the Gaussian kernel used in this study [35], was used to map the abstracts into a high dimensional space such that the positive and negative support vectors became separable by a straight line, which is known as a hyperplane in the multi-dimensional space (see right of Figure 6). In order to predict the classification of an unlabeled article (see yellow star symbol in Figure 6), the SVM model will project its abstract to the high dimensional space and classify the article according to the side of the hyperplane which its abstract falls onto.

The SVM model was also able to compute a confidence value for the classification of an unlabeled article as a 'useful' one. This can be used to rank the articles such that those with higher confidence values were shown to the evaluators first.

### General automated system

A general automated system was developed using the training set and the set of general predictors. General predictors were used because they are non-specific to TNF-α blockers and thus may improve the generalizability of the system compared to a system developed using all available predictors. The testing set was used to guide parameter selection for the modeling algorithms. The validation set was used to validate the performance of the general automated system and the generalizability set was used to assess its generalizability.

### Specific automated system

In this study, the feasibility of creating a separate automated system for each drug class or each new risk assessment task during routine risk assessment work was also investigated. Evaluators would manually classify a small number of articles related to a particular drug class and use their abstracts to develop the specific automated system. The system will then be able to automatically classify the remaining articles. In this study, the minimum number of abstracts required to train a model with satisfactory performance was determined by training models using 2 to approximately 100 abstracts from dataset A. The remaining abstracts in dataset A were used as the testing set. The selection of the abstracts was done by using Kennard and Stone sampling [39]. In this sampling technique, the abstracts that were most different in terms of their extracted terms from each other in dataset A were selected for training the models. The models were then validated using the validation set to assess their prediction performance.

### Measuring prediction performance

The performances of the models were measured using area under the receiver operating characteristic curve (AUC) [40], which is frequently used to evaluate prediction models in the biomedical informatics field [41]. AUC quantifies the probability that the model will classify a randomly chosen 'useful' article higher than a randomly chosen 'non-useful' article. Hence, a higher AUC value would indicate better performance of the model.

Lift charts were created for the general automated system. A lift chart is a graphical representation of the ranking efficiency of a prediction model. The ranks of the articles were determined using the confidence values provided by the general automated system for each unlabelled article. A good prediction model will be able to give higher ranks to 'useful' articles than 'non-useful' articles.

## Competing interests

The authors declare that they have no competing interests.

## Authors' contributions

XTW carried out the acquisition of data, categorized articles into 'useful and 'non-useful', developed automated systems, analyzed and interpret the results, and drafted the manuscript. YK took part in the study design, categorized articles into 'useful and 'non-useful', and proposed critical revisions to the draft manuscript. CWY took part in the study design, guided the development of automated systems, analyzed and interpret the results, and proposed critical revisions to the draft manuscript. All authors read and approved the final manuscript.

## Pre-publication history

The pre-publication history for this paper can be accessed here:

http://www.biomedcentral.com/1472-6947/12/13/prepub

## Supplementary Material

Additional file 1 **Appendix 1**. Classification algorithm to categorize articles into 'useful' and 'non-useful', **Appendix 2**. List of general predictors Click here for file
