# Supplementary material for: Automated systems to identify relevant documents in product risk management
Source: BMC Med Inform Decis Mak. 2012 Mar 2;12:13. doi: 10.1186/1472-6947-12-13 (PMC3315431; doi:10.1186/1472-6947-12-13)
Supplement: Additional file 1 — Appendix 1. Classification algorithm to categorize articles into 'useful' and 'non-useful', Appendix 2. List of general predictors [file 1472-6947-12-13-S1.DOC]

**Appendix 1: Classification algorithm to categorize articles into ‘useful’ and ‘non-useful’**


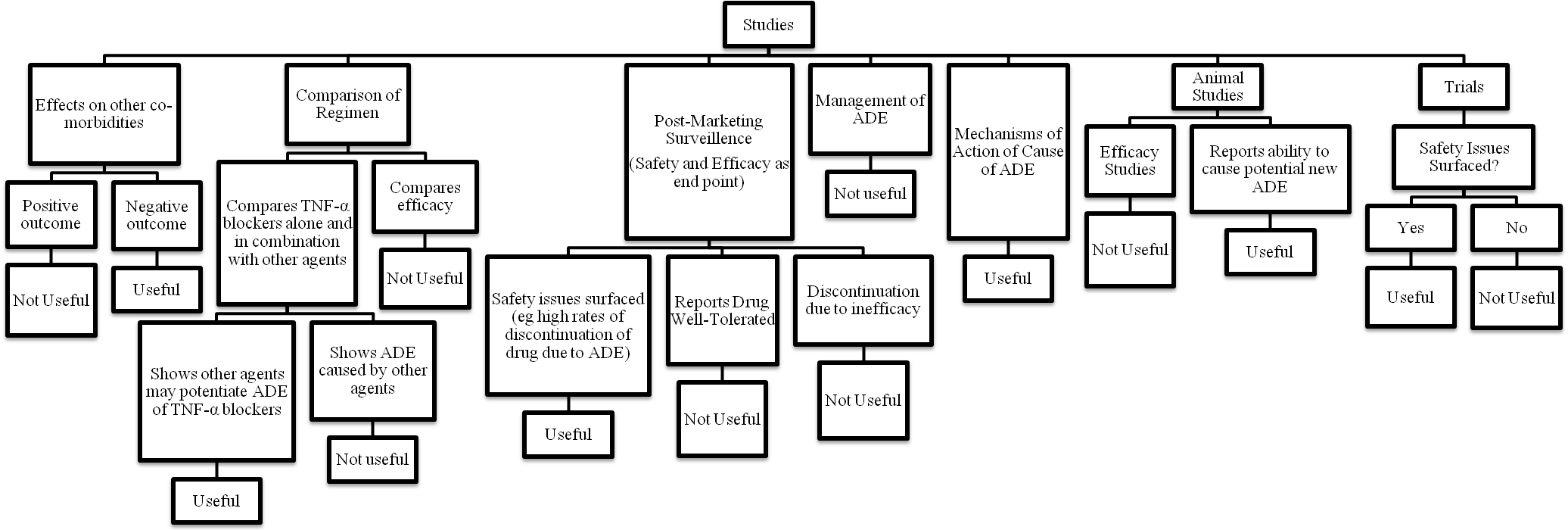


**Appendix 2: List of general predictors**

ability

abracadabra

abreast

absence

accept

accompany

accomplish

accord

account

accumulate

acquisitive

act

action

active

acute

add

adhere

adjust

administer

admit

adult

advance

adverse

affect

age

agent

aggress

agree

aid

aim

air-condition

alcohol

allow

almost

alone

already

also

alternate

although

among

analog

analysis

animal

another

antagonist

antibody

antirheumatic

apparent

appear

applicant

approach

appropriate

approve

approximate

artery

article

aspect

assay

assess

assign

associate

attenuate

attribute

author

autopilot

autopsy

average

avoid

axis

back

backboard

base

basis

bath

became

become

begin

benefit

beside

beyond

bilateral

bind

biology

blind

body

c-terminus

calculate

candidate

capacity

care

carry

cascade

case

cause

caution

cell

center

certain

cessation

chain

challenge

change

character

chart

chest

child

choice

choke

ci

circulate

class

classify

clear

clinic

close

cognizant

cohort

collect

college

combine

common

compare

complicate

component

compose

comprise

concentrate

concept

concern

conclude

concomitant

conduct

confident

confirm

consecutive

consequent

consider

consist

contain

continue

contrast

contribute

control

convention

correlate

cost

cost-effective

could

count

course

criterion

critic

damage

database

dataset

date

day

decade

december

decision

decision-maker

decline

decrease

define

degree

delay

deliver

demography

demonstrate

depend

derive

describe

design

despite

destroy

detail

detect

determine

develop

diagnose

die

differ

differential

difficult

disabled

discontinue

discuss

disease

disprove

distinct

document

dose

double

double-blind

double-label

drain

drama

drug-induced

due

dysfunction

early

effect

efficacy

eight

either

elevate

eligible

elucidate

embryo

emerge

employ

encourage

end

endothelial

endpoint

enhance

enroll

enter

enzyme

episode

equal

erosion

essential

establish

estimate

etiology

evaluate

even

event

every

evident

evolve

exacerbate

examine

excel

except

exclude

exhibit

expand

expect

experience

explain

explore

expose

express

extend

extent

eye

fail

faint

far

favor

fda

feature

few

final

find

first

five

fluid

focus

follow

follow-up

food

form

four

frequency

frequent

fulfil

full

full-length

function

furthermore

fusion

future

gain

gene

general

genetic

genotype

germline

given

globe

goal

good

great

group

guideline

halftime

hand

haq

heal

health

help

high

high-dose

higher

histology

history

hospital

hour

however

hypothesis

identify

idiopathic

image

immediate

immunomodulatory

impact

impair

implicate

import

improve

in vitro

in vivo

inadequacy

incident

include

independent

index

indirect

individual

induce

ineffective

infect

infiltrate

inflammation

inform

initial

injure

insight

insignificant

insufficient

integral

intense

interact

interest

interfere

intern

interval

intervene

intestine

intolerant

introduce

investigate

involve

isolate

issue

january

joint

key

kg

know

knowledge

lab

large

last

late

lead

league

left

less

life

like

limit

link

literature

little

local

long-term

longer

longstanding

lose

low

lumen

lung

lymphoma

m

magnet

main

maintain

major

make

male

manage

manifest

mark

marker

material

matrix

may

mean

measure

mechanic

median

mediate

medical

meet

membrane

metabolic

meter

method

mg

might

mild

minimal

minor

ml

modal

model

moderate

moderate-to-severe

modify

modules

molecule

monitor

month

morbid

moreover

mortal

mouse

much

mucosa

multicenter

multivariate

must

n

nation

nature

necessary

need

negate

neither

neutral

new

nine

non-responder

none

normal

note

novel

now

number

numeral

observe

occur

offer

often

old

one

onset

open-label

optimal

option

order

origin

outcome

outpatient

overall

p

paper

parallel

parameter

part

partial

participant

particular

pathology

pathophysiology

pathway

patient

pattern

peptide

percent

perform

period

persist

perspective

pharmacokinetic

pharmacology

phase

physician

pivot

place

placebo

placebo-controlled

placebo-treated

plasma

play

plus

point

poor

populate

posit

possible

potency

potential

practice

predict

predominant

prefer

preliminary

prescribe

presence

prevalent

prevent

previous

primary

probable

procedure

process

produce

product

profile

prognosis

progress

proliferate

prolong

promise

promote

property

proportion

propose

prospect

protect

protocol

prove

provide

publish

purpose

qsar

quality

quantity

question

questionnaire

random

range

rapid

rare

rat

rate

rather

ratio

ration

reach

reaction

reason

receive

recent

recipient

recognition

recombines

recommend

record

recover

recur

reduce

refer

reflect

refractory

regard

regimen

register

regress

regulate

relapse

relate

release

relevant

remain

remark

remission

repeat

report

represent

require

research

resolve

respect

respire

respond

result

retrospective

reveal

reverse

review

revolutionize

right-of-way

risk

role

routine

safe

safety

sample

schedule

score

screen

search

secondary

seem

seen

select

sera

series

serious

serum

seven

sharp

short-term

should

shown

side-effect

signal

similar

since

singly

site

site-specific

six

slight

slow

soluble

special

specimen

stabilize

stage

stain

standard

start

stop

strategy

study

subgroup

subject

subsequent

subset

substantial

succeed

suffice

suggest

summarize

superior

support

surface

survey

survive

susceptible

sustain

switch

swollen

symptom

synthesis

system

take

taper

target

technique

ten

tender

term

therapy

thereafter

therefore

think

third

though

three

throughout

thus

titer

together

tolerize

tool

topic

total

toward

toxic

tradition

treat

trend

trial

trigger

twelve

twice

two

typic

unchanged

unclear

uncontrolled

undergo

understand

undertake

universe

unknown

unresponsive

unusual

upon

us

use

usual

utility

valid

value

vary

variably

variant

variety

various

vein

versus

via

visit

warrant

week

weight

well

well-tolerated

whereas

whether

whose

width

will

withdraw

within

worsen

would

year-old

yet

youth

yr
